# Efficient strategies for controlled release of nanoencapsulated phytohormones to improve plant stress tolerance

**DOI:** 10.1186/s13007-023-01025-x

**Published:** 2023-05-15

**Authors:** Jimmy Sampedro-Guerrero, Vicente Vives-Peris, Aurelio Gomez-Cadenas, Carolina Clausell-Terol

**Affiliations:** 1 Departamento de Biología, Bioquímica y Ciencias Naturales, Universitat Jaume I, 12071 Castelló de la Plana, Castellón Spain; 2 Departamento de Ingeniería Química, Instituto Universitario de Tecnología Cerámica, Universitat Jaume I, 12071 Castelló de la Plana, Castellón Spain

**Keywords:** Bioactive compounds, Exogenous treatments, Plant adaptation, Release mechanisms

## Abstract

Climate change due to different human activities is causing adverse environmental conditions and uncontrolled extreme weather events. These harsh conditions are directly affecting the crop areas, and consequently, their yield (both in quantity and quality) is often impaired. It is essential to seek new advanced technologies to allow plants to tolerate environmental stresses and maintain their normal growth and development. Treatments performed with exogenous phytohormones stand out because they mitigate the negative effects of stress and promote the growth rate of plants. However, the technical limitations in field application, the putative side effects, and the difficulty in determining the correct dose, limit their widespread use. Nanoencapsulated systems have attracted attention because they allow a controlled delivery of active compounds and for their protection with eco-friendly shell biomaterials. Encapsulation is in continuous evolution due to the development and improvement of new techniques economically affordable and environmentally friendly, as well as new biomaterials with high affinity to carry and coat bioactive compounds. Despite their potential as an efficient alternative to phytohormone treatments, encapsulation systems remain relatively unexplored to date. This review aims to emphasize the potential of phytohormone treatments as a means of enhancing plant stress tolerance, with a specific focus on the benefits that can be gained through the improved exogenous application of these treatments using encapsulation techniques. Moreover, the main encapsulation techniques, shell materials and recent work on plants treated with encapsulated phytohormones have been compiled.

**Supplementary Information:**

The online version contains supplementary material available at 10.1186/s13007-023-01025-x.

## Introduction

Climate change is defined as long-term variations in global climate patterns. The increase in human activities such as deforestation, industrialisation, rapid urbanisation and the unconscious use of non-biodegradable products, produce serious contamination in the environment, which in turn has a significant impact on the climate. The extreme weather, desertification, flooded soils and the decrease in water resources cause soil instability, altered vegetation, flowering defects, pathogen defense vulnerability, and decreased agricultural productivity, leading to problems in maintaining quality crops [1]. Therefore, the negative effect of these changes decreases the capacity to meet the high food demand of the world population [2, 3].

Biotic and abiotic stresses caused by climate change increase pressure for plants [4]. Plants respond to stresses in different ways: change in gene expression, variation of growth rates, alteration in cellular metabolism, production of molecular chaperones and reactive species scavengers, etc. [5]. Among these responses, increased biosynthesis of secondary metabolites with a protective function has an important role [6]. These compounds help the plant to tolerate the adverse condition as an adaptive defense but, if the magnitude of the stress is too high or it appears too fast, they may not be enough to protect the plant completely. These metabolites are produced by plants as a defense mechanism; however, they can also be chemically synthesized or obtained from microbial sources [7, 8], and their exogenous application (via foliar or soil) can become a tool for mitigating the adverse effects of environmental stresses on plants [9]. These compounds include different acids, flavonoids and carotenoids and unsaturated fatty acids, among others (Additional file 1: Fig. S1).

It is important to highlight the role of phytohormones (PHs) as regulators of plant development and plastic growth [10]. PHs also modulate several physiological processes in plants subjected to stress conditions, and their interactions allow reconfiguring plant architecture, enhancing its capacity to adapt to negative scenarios [11]. This review emphasizes the importance of PHs in environmental stress tolerance and the benefits of exogenous hormonal treatments on plants, especially when PHs are encapsulated.

## Phytohormone modulate plant tolerance to several stresses

**Fig. 1:**
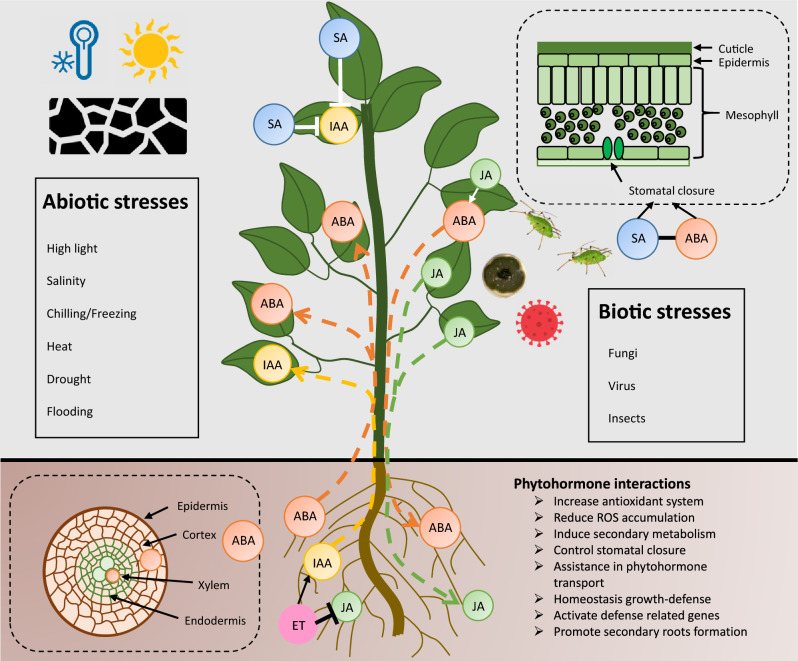
Phytohormone interactions play a crucial role in plant responses to biotic and abiotic stresses. Under biotic stress, the interaction between salicylic acid (SA) and abscisic acid (ABA) regulates stomata opening, while jasmonic acid (JA) induces ABA transport from leaves to roots. During abiotic stress, ABA is synthesized in roots and transported through the xylem, while SA blocks indole-3-acetic acid (IAA) to balance growth and defense, and ethylene (ET) inhibits JA to promote IAA synthesis and transport from roots to leaves

PHs are signalling molecules with a controlled homeostasis that mediate plant responses to internal and external stimuli [12]. They can act at their synthesis site or be transported to different parts of the plant. PHs regulate cell division, root and shoot elongation and differentiation, seed germination, dormancy, sex determination, and flowering and fruiting differentiation. Actually, the existence of different hormonal groups has been widely reported, including salicylic acid (SA), jasmonic acid (JA), abscisic acid (ABA), indole-3-acetic acid (IAA), ethylene (ET), gibberellins (GAs), cytokinins (CKs), brassinosteroids (BRs), strigolactones (SL), etc. [13, 14]. Undoubtedly, SA, JA, ABA, IAA, GA and CKs have a key role in the modulation of physiological and molecular responses to environmental stresses. The effects of phytohormones on plant development and growth, as well as their interactions under various stress conditions, are briefly discussed below and illustrated in Fig. 1:(i) SA is a phenolic compound that is principally synthesized by the phenylalanine pathway and secondarily by the isochorismate route [15]. SA promotes defense responses against pathogenic organisms and abiotic stresses such as chilling, drought, heat, heavy metals and salinity. SA controls several aspects of plant development, including seed germination, root differentiation and growth, photosynthesis, stomatal closure, senescence, flowering, and fruit yield [16]. Interestingly, SA enhances plant antioxidant capacity at low concentrations but causes pleiotropic effects and susceptibility to abiotic stresses at highest ones [17, 18]. It plays a key role in inducing the systemic acquired resistance to various pathogens and, in coordination with ABA, regulates plant defense responses against pathogens and pests [19]. When defense responses are activated, SA levels and signaling increase, leading to a reduction in auxin biosynthesis and transport. This coordination between defense and growth trade-offs helps the plant to effectively manage its resources [20]. (ii) JA, its precursor 12-oxophytodienoic acid (OPDA), and the conjugated molecules methyl jasmonate (MeJA) and jasmonoyl-isoleucine (JA-Ile), known as jasmonates (JAs), are crucial for plant development and can act directly or indirectly in defense responses [21, 22]. High concentrations of JAs are found on root tips, shoot apex, immature fruits and young leaves [23]. JAs are involved in physiological and molecular responses which protect plants against pathogenic attack, chilling, drought and high salinity. Some of the responses observed include an activation of the antioxidant system, the accumulation of amino acids such as methionine, and the regulation of the stomatal closure [24]. The interaction between JAs and ABA can have both synergistic and antagonistic effects in inducing plant tolerance. Additionally, the interaction between JAs and ET is regulated through antagonism in response to abiotic stresses [22]. (iii) ABA is an isoprenoid with an essential role in plant adaptation to abiotic stresses; among other roles it modulates stomatal opening to prevent water loss when plant suffers drought [25]. ABA is synthesized via the mevalonic acid-independent pathway and its biosynthesis starts in plastids and is carried in direction to the cytosol [26]. It also plays a role on seed dormancy and maturation, fruit ripening, and root architecture organization [27]. It is well-known that ABA improves stress responses, activating stress-related pathways and modifying gene expression [28, 29]. It also regulates cell turgor and restricts cell growth as adaptation mechanisms [30]. In plants exposed to abiotic stresses, ABA interacts with auxins to control root meristem activity and lateral root development [31]. (iv) IAA is the most studied auxin and has been reported as a vital molecule for the proper development of plants [32]. It promotes cell division, differentiation and elongation, after plants exposure to stress. Auxins activate numerous genes in response to abiotic and biotic stress responses, although their role as a stress response regulator is still under study [33]. It is known that the crosstalk between IAA and SA mediates plant tolerance [34]. However, when plants are subjected to multiple stresses simultaneously, their homeostasis is altered, leading to changes in genes related to auxin transport, such as *PIN1*. This can result in the inhibition of IAA transport in the plant [35]. Excessive IAA accumulation causes altered morphogenesis of principal root and avoids the formation of lateral roots, disrupting the nutrient uptake [36]. (v) GAs are a group of molecules derived from a tetracyclic diterpenoid carboxylic acid that has positive effects on tissue expansion, trichome initiation, and the development of flowers and fruits [37]. There is also evidence that GAs play a role in abiotic stress adaptation, where their antagonistic interaction with CKs helps control the elongation of the plant shoot apex and root tip [38]. (vi) CKs control chloroplast differentiation, cell division and interaction with other organisms (especially pathogens) in the plant. Interestingly, plants alter their endogenous CK levels in response to abiotic stress (heat and chill) [39].

PHs have been extensively used as exogenous treatments for enhancing plant tolerance to both biotic and abiotic stresses, with numerous studies highlighting their potential to improve plant growth, development, and stress responses, as shown in Table 1. Traditional methods to treat plants with PHs consist in either adding them to a nutrient solution for root absorption or spraying them to the aerial organs. Among them, the use of absorbent cotton to maintain the concentration of the phytohormone and promote a correct absorption by the plant is one of the most popular [110]. Plants absorb PHs through leaf stomata or rhizodermis, to later transport them to the internal structures by ion channels and protein transporters, through phloem and xylem [111]. PHs are recognized by specific protein receptors inside plant cells. For instance, SA joins to NON-EXPRESSER OF PATHOGENESIS-RELATED GENES 1 (NPR1), JA joins to CORONATINE INSENSITIVE 1 (COI1), ABA joins to PYRABACTIN RESISTANCE1/PYR1-LIKE (PYR/PYL), IAA joins to TRANSPORT INHIBITOR RESPONSE 1 (TIR1), ET joins to ETHYLENE RECEPTOR 1 (ETR1), GAs join to GIBBERELLIN-INSENSITIVE DWARF 1 (GID1), CKs join to CYTOKININ RESPONSE 1 (CRE1), BRs join to BRASSINOSTEROID INSENSITIVE 1 (BRI1) and SL joins to DWARF 14 (D14) [112]. However, exogenous applications of free PHs have several problems such as the difficulty to define the correct dosage. Depending on the application purpose and chosen technique, the plant might need different doses, ranging from low quantities (at the nanomolar level) to much higher amounts, which is costly and inefficient. Furthermore, externally applied products are expected to maintain their initial concentration in PHs and be stable over time but diverse environmental conditions and low stability of the molecules can affect the treatment. Even the structure of the molecule can be affected by light or temperature, modifying its behaviour and decreasing its efficiency [113].

**Table 1:** Representative list of phytohormones applied in treatments to improve the resistance of plants to abiotic and biotic stresses

Phytohormones	Plants	Stresses											**References**
		Abiotic								Biotic			
		Heat	Chilling	Drought	Salinity	Heavy metals (Cd, Cu, Ni, Pb)	Freezing	Osmotic	UV	Bacteria	Fungi	Insect, nematode or virus	
Salicylic acid	Tomato	✔	✔	✔	✔						✔		[40–42]
	Bean	✔	✔	✔		✔							[40, 43]
	Maize	✔		✔	✔	✔							[44–47]
	Barley		✔	✔		✔		✔					[48–51]
	Wheat	✔	✔			✔							[52–54]
	Rice			✔		✔			✔	✔	✔		[55–59]
	Orange										✔		[60]
	Banana		✔								✔		[61, 62]
	Tobacco											✔	[63]
	Arabidopsis					✔					✔		[64, 65]
Jasmonic acid	Tomato	✔			✔								[66, 67]
	Maize			✔		✔							[68, 69]
	Rice	✔	✔	✔	✔							✔	[70–73] [74]
	Orange		✔										[75]
	Banana		✔										[76]
	Soybean				✔	✔							[77, 78]
	Canola										✔		[79]
	Arabidopsis			✔			✔			✔	✔		[80–83]
Abscisic acid	Tomato			✔									[84]
	Maize			✔									[85]
	Rice									✔	✔		[86, 87]
	Wheat			✔									[88]
	Cucumber		✔										[89]
	Bean										✔		[90]
	Arabidopsis									✔			[91]
Indole acetic acid	Tomato					✔							[92]
	Pea				✔								[93]
	Alfalfa			✔									[94]
	Maize				✔								[95]
	Wheat				✔								[96]
	Soybean			✔									[97]
	Potato				✔								[98]
Gibberellins	Tomato					✔						✔	[92, 99]
	Maize				✔								[95]
	Rice											✔	[100]
	Wheat				✔								[101]
	Potato				✔								[98]
	Fava bean					✔							[102]
	Soybean					✔							[103]
Cytokinins	Arabidopsis		✔							✔			[104, 105]
	Cucumber		✔										[106]
	Rice	✔								✔			[107–109]

Exogenous application of PHs can have negative biological impacts in plants. Firstly, hormonal imbalances may arise from excessive application, which can affect normal plant growth and development and increase plant susceptibility to pests [114]. Therefore, PHs can alter plant morphology by inducing the formation of adventitious roots or altering leaf shape; excessive use of GAs can lead to weakened stems and increased susceptibility to pests and diseases. In this sense, in citrus trees, over-saturation of uptake capacity due to GA applications can lead to the production of small fruits with poor flavour [115]. Secondly, long-term PHs treatments can cause plants to become dependent on external PH sources, leading to a loss of their natural ability to generate hormones, which can adversely affect growth rate and health [116]. From an ecological point of view, the application of PHs can have also some negative effects. In the case of treatments applied to the watering solution, a large amount of a free PH could affect the microbiological communities associated with the plant, changing soil ecosystem characteristics and even altering nutrient levels [117]. Moreover, some plant hormones, such as synthetic auxins, can have negative impacts on non-target organisms like pollinators. In this way, the herbicide 2,4-D, which consists in a synthetic auxin, has been shown to harm bees and other beneficial insects [118]. Excessive or inappropriate use of plant hormones can lead to contamination of soil and water. In addition, the use of synthetic growth regulators like paclobutrazol in crop production has been shown to affect the health of organisms and ecological systems [119]. It is important to note that the ecological impacts of PHs applications depend on the specific hormone being used, the method and timing of application, and the surrounding ecosystem. As such, it is important to carefully consider the potential risks and benefits of any plant hormone application. Encapsulation can help mitigate these issues by allowing for better management of PHs application and dosage.

## Encapsulation can improve phytohormone biological effects in agriculture

**Fig. 2:**
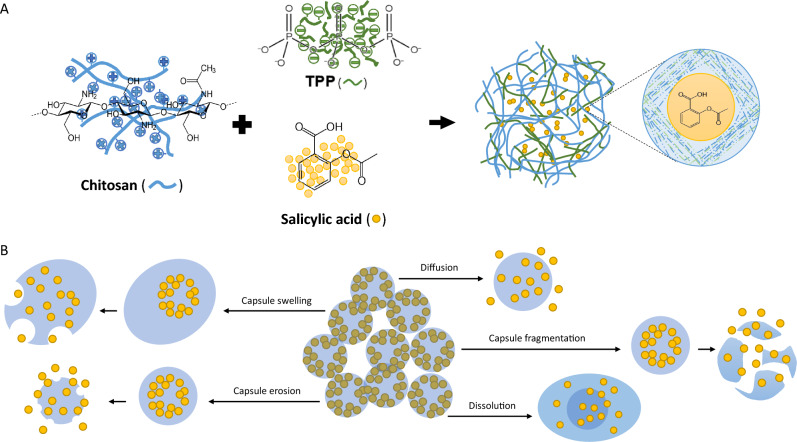
General processes of encapsulation and release of the active ingredient. Graph **A** represents SA encapsulation using chitosan as shelling material and tripolyphosphate (TPP) as bridge to form the nanocapsule. Graph **B** represents different mechanisms of PHs released from shell

Encapsulation has attracted attention due to the possibility of controlled release of most biologically active compounds and for the eco-friendly nature of the biomaterials used as coatings [120]. Encapsulation produces particles with high hydrophilicity and lipophilicity, enhancing their ability to penetrate plant tissues [121]. This is a process where a bioactive compound or active agent, defined as core material, is packaged or coated in a carrier (protective material) to create capsules with enhanced biological characteristics (Fig. 2A). The coating material is used to encapsulate the bioactive compounds forming a matrix capable to create a barrier for the core against important factors such as: heat, oxygen concentration, light, pH and shear [122]. Capsules are able to inhibit volatilization and protect the core versus extreme environmental conditions, reducing its sensitivity to degradation [123]. Encapsulation is an effective alternative to solve physical or chemical instability problems of PHs. These kind of compounds are encapsulated for increasing their durability and functionality, in addition to obtain a controlled release [124]. For a successful encapsulation, it is important to consider and correctly select three factors: (a) the core, target active agent to encapsulate, (b) the shell, coat or wall material used as coating and, (c) the encapsulation method, depending on the nature of the materials and the final application [125]. Plant treatments performed with encapsulated PHs have increased in recent years due to their ability to promote plant growth and control the pathogen effects [126].

### Principal encapsulation techniques used in agriculture

The selected encapsulation methodology depends on the core and shell characteristics, and their chemical and physical properties. The chosen technique has the challenge to achieve a high encapsulation efficiency and a controlled release capacity [127]. During the encapsulation process, the active agent must remain intact, and the coating should not exhibit adherence or aggregation. The newly formed particles must have a homogeneous particle size distribution, with particles free of dents and/or holes [128]. Before starting the encapsulation process, the physical state of the core (solid or liquid) divides the fabrication process in either coating the solid particles with the shell material in a pan coater or fluidized bed, or forming droplets using an immiscible liquid or air, followed by droplet solidification [129]. The coat shell (in general, the capsules), can take numerous morphologies that could be classified based on the size of the encapsulation, into: nanocapsules (diameter < 0.2 μm), microcapsules (diameter between 0.2 and 5000 μm), and macrocapsules (diameter > 5000 μm) [130, 131]. Moreover, capsules can be divided into microcapsules and microspheres, depending on their shape and construction. While microcapsules have a central inner core, which contains the active compound, in microspheres the core is heterogeneously dispersed in the encapsulation material. In general, encapsulation techniques fall into three categories: chemical, physical–chemical and physical–mechanical approaches. Table 2 shows the most important techniques used to encapsulate phytohormones, as well as their advantages and disadvantages.

**Table 2:** Different encapsulation techniques used in agriculture to form capsule-core samples

Encapsulation techniques						
Process	Chemical			Physico-Chemical		
	Ionic gelation	In-situ polymerization	Liposome entrapment	Coacervation	Sol–gel encapsulation	Solvent evaporation
Diagram						
Advantages	• Simple process •High encapsulation efficiency	• Inexpensive materials • Simple manufacturing	• Stable	• Versatile operation • High encapsulation capacity	• Good thermal stability • Good encapsulation efficiency	• Simple procedure • Low cost
Disadvantages	• Limited polymers • Produced always in aqueous dispersion	• Complex procedure • Use a toxic precursor	• Difficult to scale	• Expensive process • Agglomeration • Difficult to scale up	• Long process time • Use of toxic organic solutions	• Low efficiency encapsulation • Restricted process
PHs encapsulated	SA, IAA, GAs	SA, ABA, GAs	SA, CKs	JAs	ABA	JAs
Particle size range	0.5–1000 μm	0.05–1100 μm	2–1200 nm	2–1200 μm	0.2–20 μm	0.5–1000 μm
Shelf life	Short	Short	Short	Short	Medium	Poor
Reliability	Poor	Poor	Poor	Poor	Good	Poor
References	[129, 132, 133]	[129, 132, 134]	[129, 132, 135]	[129, 132, 136]	[129, 132, 137]	[129, 132, 138]

Process	Physical–Mechanical					
	Spray drying	Multi-orifice centrifugation	Pan coating	Co-extrusion	Fluidized bed	Air suspension coating
Diagram						
Advantages	• Simple process • Easy to scale up • Adjustable cost	• Can use solid and liquid core materials	• Low cost	• Stability • High retention	• Extensive capsule materials	• Low cost
Disadvantages	• Limited capsule materials • High energy consumption	• High temperatures required	• Inconsistent encapsulation efficiency • Difficult to control • Time-consuming	• Limited capsules materials	• Restricted to solid particle coating • Agglomeration	• Restricted to solid cores • Complex process
PHs encapsulated	SA, ABA	ABA	SA	JAs, IAA	ABA	SA
Particle size range	5–5000 μm	5–1500 μm	600–5000 μm	500–3000 μm	20–1500 μm	0.1–1000 μm
Shelf life*	Large	Medium	Short	Medium	Medium	Medium
Reliability**	Good	Good	Poor	Good	Good	Poor
References	[129, 132, 139]	[129, 132, 140]	[129, 132, 141]	[129, 132, 142]	[129, 132, 143]	[129, 132, 144]

^*^Shell life: large (30–60 weeks), medium (10–30 weeks) and Short (< 10 weeks)

^**^Reliability refers to degradation of encapsulated at temperature gradient (Thermogravimetric analysis): poor (> 50% of mass lost) and good (< 50% of mass lost)

The principal chemical techniques are: (i) ionic gelation, which synthesizes particles from electrostatic interactions of ions with opposite charges. This technique requires a polymer (as chitosan or alginate), a crosslinker, generally sodium triphosphate (TPP), and constant conditions of mechanical stirring [133]; (ii) *in-situ* polymerization consists in adding a biomolecule (core) to a polymer solution (shell material) and dispersed it until a certain size is obtained. Polymerization is performed in the continuous phase with no reactants added to the core material [134] and (iii) liposome entrapment, in which a lipid-based encapsulation system is used as a carrier for active compounds such as antioxidants, hormones, peptides, etc. This system is widely used due to its lipophilic/hydrophilic and compartmentalization properties [135]. In the case of physical–chemical techniques, there are mainly three: (i) coacervation, a process that involves the electrostatic attraction between two polymers with opposite charges and coacervate formation by pH changes, which generally consists of four steps: (a) suspension of the core in a liquid phase, (b) addition of the polymer solution around the core, (c) gelation, and (d) solidification of the capsule wall [136]; (ii) sol–gel encapsulation, in which an emulsion is produced from two immiscible phases prepared in the presence of a surfactant agent. Silica (Si) based particles are the most widely used because it is possible to obtain Si particles with a specific size and shape by changing the pH of sol–gel materials [137] and (iii) solvent evaporation, where a polymer is dissolved in an organic solvent, and then dispersed in an aqueous solution (with the core material) to form an emulsion, using a surfactant agent. Once the emulsion is formed, the organic solvent must be evaporated to obtain the final particles [138].

Concerning physical–mechanical techniques, the following are highlighted: (i) spray drying, a fast and scalable process that allows obtaining dry powders from liquid suspensions [139]. Briefly, the suspension is sprayed through a nozzle, using a hot gas (either air or nitrogen), generating solid particles that move with the air stream and are collected by a cyclone [145]; (ii) multi-orifice centrifugation, is a process that launches the core through a counter-rotating disk using centrifugal force [140]. The core passes through a membrane composed of the shell material, forming the encapsulated particles [146]; (iii) pan coating, is a method in which a coating composition is added to a moving bed of core material using hot air to evaporate the solvent. The core material rotates on a pan while the coating material is applied at the same time [141]; (iv) co-extrusion, consists of mixing the material of the core with that of the shell by means of a system of nozzles. The vibrations produced are capable of breaking the liquid phase and forming drops, which become capsules when falling into a solidification bath [142]; (v) fluidized bed, this process is performed by spraying a shelling solution into a fluidized bed with the core, requiring numerous wetting–drying cycles to form a continuous film [143]; and (vi) air suspension coating, in this technique the core is suspended in an upward draft and continuously coated with sprayed shell material [144]. The core passes through the coating-zone cyclically until it is encapsulated. The air stream allows in turn to dry the encapsulated particles [147].

### Principal coating materials used in agriculture

The coating material influences the controlled release of bioactive molecules, also affecting their bioaccessibility. It is important that the shell or coating is not reactive or produces a non-specific conformation on its own. The chosen materials must provide, mainly, protective properties, in addition to others such as flexible structure, stability, strength and permeability [148]. The initial core/shell ratio and the amount of shell are essential parameters during the encapsulation process since they directly affect the dispersion process and determine the particle surface area under specific conditions [149]. In relation to the environment, it is required that the coating material be also inert (does not react with the active principle). Its surface must be flexible to encapsulate and release different compounds, but also strong to protect against extreme conditions and, after use, it must be biodegradable to minimize environmental impact [150]. One of the main considerations is the shelling material structure, since it determines the capsule functional properties. The ideal shell material should have a stable emulsifying property and an easy handling during the encapsulation process. Furthermore, it must preserve its permeability and not react with the core during long-term storage conditions [151]. It must be soluble in several solvents and, at high concentrations, the rheological properties under the influence of stresses must be stable, but with a desired flexibility that does not compromise its structure [152]. In some cases, to enhance the shell properties, the use of a combination of coating materials is necessary.

There are many different materials used for the encapsulation process, for example: polysaccharides from different sources (plant, marine algae and fungi), lipids, proteins, synthetic or inorganic, and waxes, among others. Recent trends in agriculture aim to use inert or biodegradable matrices for encapsulating plant extracts (flavonoids, fatty acids and main phytohormones) [153]. Polysaccharides are by far the most widely used shell material due to their structure, abundance and biodegradability [154]. Alginate, a natural hydrophilic compound isolated from algae cell wall, is widely used to formulate films, hydrogels, microspheres and microcapsules, since this material exhibits important shelling characteristics, such as moisture absorption, gelation and biocompatibility [155, 156]. Chitosan is undoubtedly the most popular coating material due to its superior characteristics, such as biocompatibility, biodegradability, resistance, non-toxicity and its ability to form films without relying on additives [157]. In agriculture, chitosan encapsulated molecules are used as an economic and ecological alternative to formulate biofertilizers, biopesticides, conditioners and growth promoters [158]. Among polysaccharides, starch has gained interest as a nanocarrier system, mainly due to its abundance, availability, biodegradability and competitive cost. In addition, starch can exhibit diverse molecular structures depending on its plant tissue origin, such as fruits, roots, seeds, and tubers. Its unique structure can result in a variety of shapes, sizes, and granule compositions [159, 160]. Gum polysaccharides (arabic, carrageenan, xanthan, among others) are used as coating materials due to their favorable characteristics, including excellent emulsification, high solubility, low viscosity, and inhibition of oxidation reactions [161].

Other interesting coating materials are amorphous silica, waxes and caseinates. Amorphous silica (SiO_2_) is a non-toxic material which use in the encapsulation process is inexpensive and its manufacture is safe and friendly to the environment [162]. This material is used to encapsulate different bioactive agents by entrapment in its inner pores, which allows a chemical and physical stabilization between the core and the shell [163]. Waxes become more relevant due to their favourable properties such as hardness, hydrophobicity, scratch resistance and thermal stability. In fact, it is interesting to carry out studies on the microstructure and properties of new waxes to control possible interactions with other components [164, 165]. Caseins are a class of milk-derived proteins, similar to whey proteins, containing casein micelles and caseinates as extended forms [166]. Caseins have the facility to form suspensions and, during capsule formulation, have the capacity to emulsify and foam [167]. Table 3 shows the principal coating materials used for encapsulation, as well as their advantages and disadvantages. While not all of these materials have been utilized for PH encapsulation, they have demonstrated efficacy for encapsulating other molecules and compounds and therefore present promising options for future applications.

**Table 3:** Main materials used to form capsule shell

Materials	Advantages	Disadvantages	Applicable stress type	References
Polysaccharides				
Alginate	• Low toxicity • Bio inert material • Low cost encapsulation process	• Limited changes on mechanical properties • Instability caused by ion-leaching	Biotic	[155, 168, 169]
Carrageenan	• Not toxic • Biocompatible • Biodegradable	• Potential reaction with bioactive molecules	Abiotic / Biotic	[170–172]
Chitosan	• Not toxic • Enhanced biocompatibility • High stability • Expensive dosing is prevented	• Method of preparation depends on the PHs used	Abiotic / Biotic	[157, 173, 174]
Gum Arabic	• Abundant availability • Excellent core protection ability	• Limited availability • High cost	Abiotic	[175, 176]
Modified starch	• Fully biodegradable • Inexpensive material • Can be easily modified	• Loose structure due to its poor resistance to shearing and stirring • Toxicity of several derivative products	Biotic	[159, 177, 178]
Maltodextrin	• Low hygroscopicity • Protect bioactive compounds from oxidation	• Poor stability • Low retention	Biotic	[179, 180]
Pectin	• Low cost encapsulation process • Possibility to modify its structure	• High swelling degree in unfavourable environments	Biotic	[181–183]
Inorganic				
Amorphous silica	• Biocompatible • High uptake capacity • Controlled drug release system • Low toxicity • Improved loading and releasing properties	• Difficult to predict successful amount of encapsulated drug	Abiotic/Biotic	[162, 184]
Synthetic and natural polymers				
Polyvinyl alcohol	• Biodegradable • Not toxic • Biocompatible	• Low stability • Chemical modification	Abiotic	[185, 186]
Polyacrylamide	• High stability	• Toxic	Abiotic	[187, 188]
Fats and waxes				
Hydrogenated vegetable oils	• Controlled release	• Multiple steps in the preparation process	Biotic	[189, 190]
Bees wax	• Highly diverse • Adaptable material to changes in different conditions • Degradable	• Low encapsulation capacity	Abiotic	[191, 192]
Paraffin wax	• Structure does not change over time	• Not adjustable • Not adoptable • Toxic	Abiotic	[193, 194]
Proteins				
Soft gelatine capsule (SGC)	• High accuracy • Reduces dustiness during manufacturing	• Expensive to produce • Not adaptable	Biotic	[195, 196]
Hard gelatine capsule (HGC)	• Rapid drug release	• Problems with cross-linking • Not suitable with hygroscopic compounds	Biotic	[195, 197]
Sodium caseinates	• Oxidative stability • Biocompatibility • Increases encapsulation efficiency	• Requires a significant amount of bioactive compound	Abiotic	[166, 198, 199]

### Release mechanisms of active ingredients

The release of encapsulated bioactive compounds can occur through controlled and uncontrolled mechanisms. The rapid release could be ineffective but, on the contrary, an extremely slow release could decrease their positive effects and cause problems in their entrance through the plant tissue surface. Controlled release requires a trigger stimulation to start. The deployment of this mechanism ensures a long-lasting action of the bioactive molecule with an expected concentration [200]. Furthermore, it is important because its manoeuvrability and predictability characteristics allow the estimation and study of the core release rate [201]. The release rate study considers several parameters such as starting point and duration, kinetics, released quantity, speed and release mechanism [202]. There are five mechanisms of release: (i) diffusion, which refers to the random movement of the core, typically caused by a concentration gradient. In this process, the release of the active agent depends on various factors, including the physical–chemical characteristics of both the core and the matrix, as well as the ratio between them [203]; (ii) swelling, where differences in solvent concentration cause the whole shell structure to swell with increased pore size, making it difficult to maintain capsule integrity and causing core molecule release [204]; (iii) fragmentation, occurs when the matrix is disrupted by physical, chemical, or biological stresses, and in this process, the amount of core released depends on the magnitude of the stress as well as the shape and size of the resulting capsule fragments [205]; (iv) erosion, a process that can be caused by various factors, including temperature, pH, enzymes, and mechanical stimulation. This process can occur in two ways: surface erosion, which involves degradation of the capsule surface, and bulk erosion, which involves degradation of the entire capsule [206]; and (v) dissolution, which refers to the release of the bioactive core into an liquid medium either through the dissolution of the matrix or without it. This process can start either on the surface of the application point or after it has been breached [207]. In the Fig. 2B, a schematic procedure of each release mechanism is depicted.

### Encapsulated phytohormone development to enhance plant stress tolerance

Plants exhibit diverse responses to stress depending on the affected area. These responses may include changes in nutrient translocation, cell death at the entrance of the affected zone, alterations in gene regulation or cell wall composition, production of lipids, metabolites, and proteins, as well as the synthesis of antioxidant compounds. [208–210]. Plant response is influenced by its genotype and stage of development, the duration and intensity of the stress, the combination of different stresses, etc. This response, which is controlled by a complex network, starts with the stress perception, triggering various molecular events that end with phenotypic, physiological, developmental and metabolic changes [211]. Despite the various response mechanisms, plants may still be vulnerable to stress when it is severe, causing both internal damage such as cell wall and DNA disruption, lipid peroxidation, protein deformation, and mitochondrial cleavage, as well as external damage such as reduced seed germination, decreased biomass, altered root growth, and pleiotropic effects [212]. These problems can be solved using encapsulated phytohormones that, through their controlled release, allow the correct internalization of the different molecules. Several studies have shown that encapsulated SA generates pathogenic resistance against *Fusarium verticillioides* and *Sclerotium rolfsii* in maize and rice, respectively [213, 214], and cold and salt tolerance in sunflower and grape [215, 216], respectively. Treatments with encapsulated JA and ABA provide resistance against cold and drought stress in cherry tomato and Arabidopsis [217, 218], respectively, and treatments with encapsulated IAA and GAs enhance plant growth and seed germination rates in tomato and bean [219, 220]. Once the plant recognizes that it is under stress, signal transduction cascades are triggered and start a fluctuation between growth and stress response [94]. Encapsulated phytohormones offer a unique advantage in that treated plants do not need to activate signal cascades or biosynthesis pathways. Instead, plants can simply take up the released and available phytohormones, which can induce controlled changes in plant growth and development. Table 4 compiles the main works where encapsulated PHs are used to promote stress tolerance and growth development.

**Table 4:** Encapsulated phytohormones used in treatments to improve stress resistance and tissue development in plants

Encapsulated phytohormone	Encapsulation method	Capsule Material	Plants treated	Agriculture benefit	References
Salicylic acid	• In-situ polymerization	• Alginate	• *Helianthus annuus L*. (Sunflower)	• Tolerance of the tissue to the cold storage	[215]
	• Spray drying	• Amorphous silica and chitosan	• *Arabidopsis thaliana*	• Reduces deleterious effect of SA on treated plants	[18]
	• Ionic gelation	• Chitosan	• Zea mays (maize)	• Control of *Fusarium verticillioides* diseases and act as biostimulant	[213]
	• In-situ polymerization	• Alginate	• *Oryza sativa* (rice)	• Control of *Sclerotium rolfsii* disease	[214]
	• Ionic gelation	• Chitosan	• *Vitis vinifera* (grape)	• Protection against salinity stress	[216]
Jasmonates	• Solvent evaporation	• Gliadin-Casein	• *Solanum lycopersicum* var. *cerasiforme* (cherry tomato)	• Used as coating to enhance cold time storage	[218]
	• Coacervation	• Alginate and chitosan	• *Solanum tuberosum* (potato)	• Tuber postharvest treatment for preserving	[221]
	• Co-extrusion	• PLGA	• *Vitis vinifera* (grape)	• Pest management	[222]
Abscisic acid	• Sol–Gel encapsulation	• Amorphous silica	• *Arabidopsis thaliana*	• Provides resistance against drought stress	[217]
	• In-situ polymerization	• Lignin	• *Oryza sativa* (rice) and *Arabidopsis thaliana*	• Increases drought resistance	[223]
Auxins	• Ionic gelation	• Chitosan	• *Solanum lycopersicum* (tomato)	• Increase germination and seedling growth rate. Acts as biostimulant	[220]
	• Co-extrusion	• Alginate and chitosan	• *Solanum lycopersicum* (tomato)	• Increase morphological characteristics	[224]
	• Ionic gelation	• Chitosan	• *Malus domestica* (apple)	• Promote adventitious rooting	[225]
Gibberellins	• Ionic gelation	• Chitosan	• *Phaseolus vulgaris* (bean)	• Promote germination of seeds and enhances plant fertility	[219]
	• Ionic gelation	• γ-PGA polymer	• *Phaseolus vulgaris* (bean)	• Increase germination rate, and leaf and root development	[226]
	• Interfacial polymerization	• Chitosan and alginate	• *Solanum lycopersicum* (tomato)	• Promote plant development and enhance fruit productivity	[227]
Cytokinins	• Liposome entrapment	• Liposomes	• *Cocos nucifera L. var Makapuno* (Coconut)	• Enhance bioactivity formation of callus in vitro	[228]

While phytohormone-loaded nanocapsules have shown promise in mitigating the harmful effects of various types of stress, including their combination, it is important to test their efficacy for each specific condition. Although studies examining the effects of two or more combined stressors have increased in recent years, the use of encapsulated PHs for mitigating multifactorial stresses remains poorly studied and requires further exploration. Given that plants in nature are often subjected to multiple negative conditions simultaneously, understanding the potential of nanocapsules in mitigating these complex stress scenarios could have significant implications for improving plant health and productivity [229]. Among the scarce literature in this issue, a recent work has explored the benefits of the application of benzenedicarboxylic acid impregned in calcium nanoparticles to mitigate the combined stress induced by the organic pollutant dichlorodiphenyltrichloroethane and cadmium in *Brassica alboglabra* plants [230]. This reveals the importance of spreading the use of nanoparticles under stress combination, where encapsulated PHs could bring new strategies in this disturbing scenario.

## Conclusions and future perspectives

New forward-thinking solutions to improve crop tolerance to extreme climatic conditions must be obtained. Today, the world demands bioactive compounds that do not affect the environment. The development of biomaterials based on nanotechnology offers new products with applications in agriculture. The encapsulation of PHs could be an affordable solution to fight against environmental stresses, reducing their negative effects on plant development and yield, without affecting other characteristics of the crop as its nutritional value.

Recent studies highlight the main role of PHs, such as SA, JA and ABA in plant responses to environmental stress. The exogenous application of PHs activates the response mechanisms that help plants to cope with nutrient deficiency and growth regulation under stress. Studies carried out in vivo and in vitro have evaluated the bioavailability and controlled release of different products, although the study of the possible interactions between the encapsulated compounds and the matrix within the formulations is still required. In addition, it is important to determine several properties of these nanocarrier systems, such as particle size, charged surface area, surface coating and solubility. These characteristics are essential because they condition the possible toxicological effects. Indeed, toxicology studies based on physical–chemical characteristics, experimental design synthesis and exposure time in the plant would allow the development of new nanocarriers with efficient applications, and those that are not hazardous for the environment and plant health.

Further studies are necessary to investigate the synergistic and antagonistic interactions of PHs within plants. This will require the use of different biotechnological approaches to identify the metabolites, signals and genes induced during PH treatments. Additionally, studying the interplay between PHs could provide new insights into their role in stress tolerance. Manipulating the endogenous levels of PHs through encapsulation and observing their response in different tissues/organs during various stresses can be an exciting tool for improving plant stress tolerance in modern agriculture. However, it is crucial to consider the interactions between the environment and plant species, as this information can be used to optimize PH behaviour, dosage, and treatment timing. In summary, a better understanding of PH interactions and their effects on plant stress tolerance requires multidisciplinary approaches, and considering the environment-plant species interactions can help us develop effective strategies for using PHs in agriculture.

## Supplementary Information

**Additional file 1: Figure S1**. General plant-derived compounds used in treatments.

## Data Availability

Data are contained within the article.
