# Supplementary material for: Efficient strategies for controlled release of nanoencapsulated phytohormones to improve plant stress tolerance
Source: Plant Methods. 2023 May 15;19:47. doi: 10.1186/s13007-023-01025-x (PMC10184380; doi:10.1186/s13007-023-01025-x)
Supplement: Supplementary file 1 — Additional file 1: Figure S1. General plant-derived compounds used in treatments. [file 13007_2023_1025_MOESM1_ESM.pptx]

## Slide 1
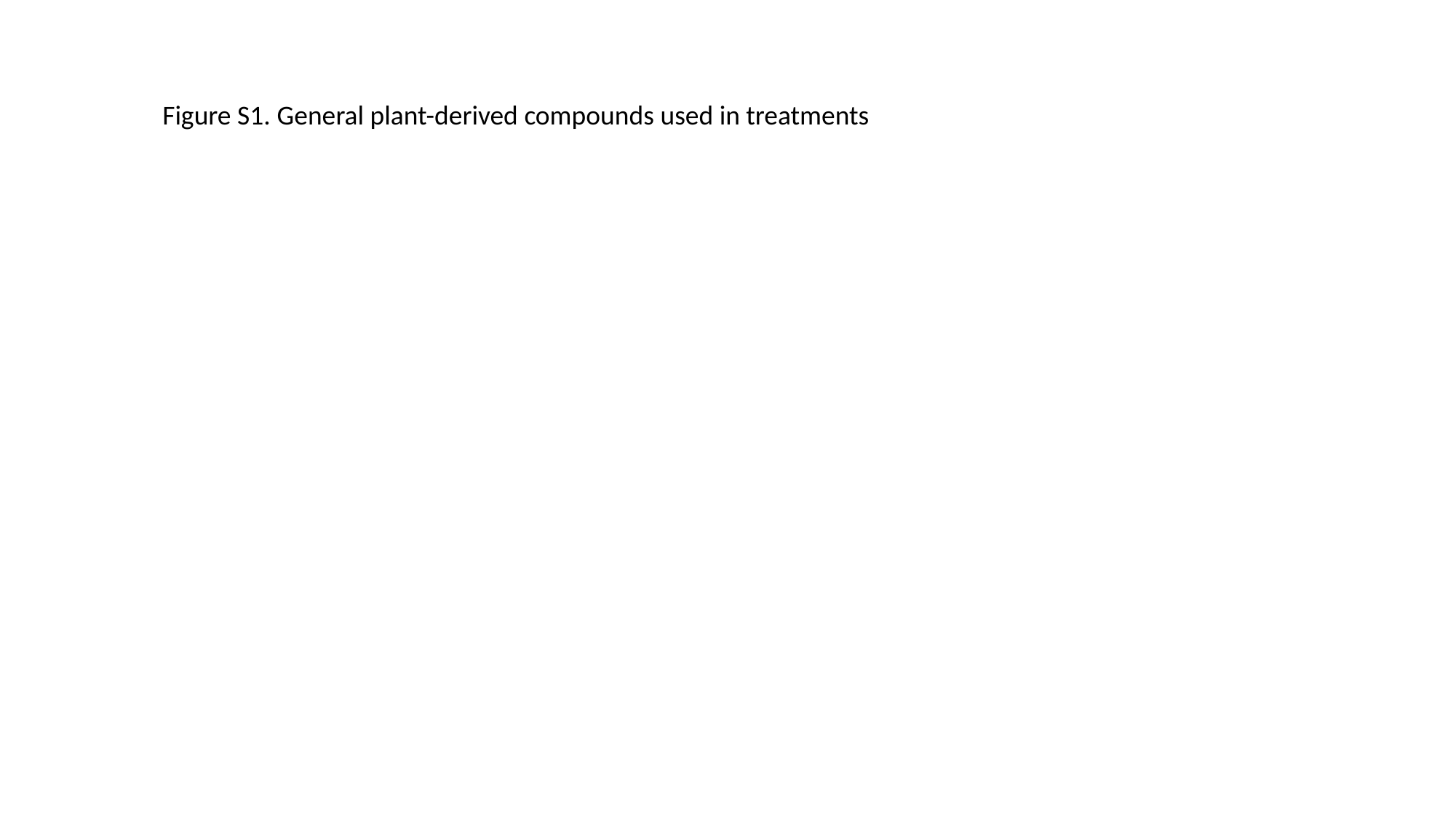

Figure S1. General plant-derived compounds used in treatments

## Slide 2
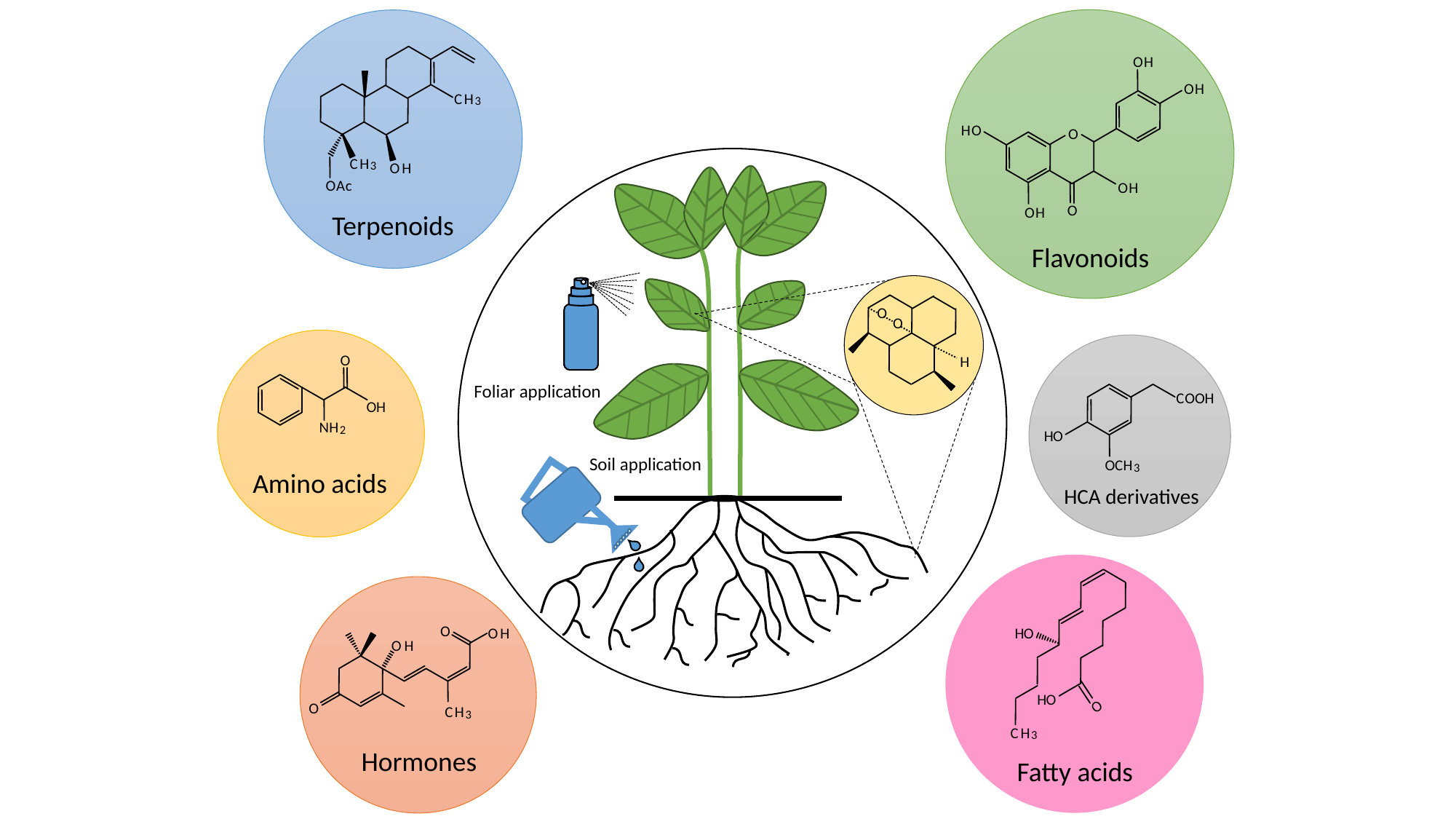

C
H
3
C
H
3
O
H
O
A
c
O
H
O
H
H
O
O
O
H
O
O
H
Terpenoids
Flavonoids
O
O
H
N
H
2
Foliar application
O
H
C
O
H
O
C
H
O
3
Soil application
Amino acids
HCA derivatives
H
O
H
O
O
C
H
3
O
O
H
O
H
O
C
H
3
Hormones
Fatty acids
O
O
H
